# Moiety modeling framework for deriving moiety abundances from mass spectrometry measured isotopologues

**DOI:** 10.1186/s12859-019-3096-7

**Published:** 2019-10-28

**Authors:** Huan Jin, Hunter N. B. Moseley

**Affiliations:** 1 Department of Toxicology and Cancer Biology, University of Kentucky, Lexington, KY USA; 2 Department of Molecular & Cellular Biochemistry, University of Kentucky, Lexington, KY USA; 3 Markey Cancer Center, University of Kentucky, Lexington, KY USA; 4 Resource Center for Stable Isotope Resolved Metabolomics, University of Kentucky, Lexington, KY USA; 5 Institute for Biomedical Informatics, University of Kentucky, Lexington, KY USA

**Keywords:** Stable isotope resolved metabolomics (SIRM), Moiety model, Isotopologue deconvolution

## Abstract

**Background:**

Stable isotope tracing can follow individual atoms through metabolic transformations through the detection of the incorporation of stable isotope within metabolites. This resulting data can be interpreted in terms related to metabolic flux. However, detection of a stable isotope in metabolites by mass spectrometry produces a profile of isotopologue peaks that requires deconvolution to ascertain the localization of isotope incorporation.

**Results:**

To aid the interpretation of the mass spectroscopy isotopologue profile, we have developed a moiety modeling framework for deconvoluting metabolite isotopologue profiles involving single and multiple isotope tracers. This moiety modeling framework provides facilities for moiety model representation, moiety model optimization, and moiety model selection. The moiety_modeling package was developed from the idea of metabolite decomposition into moiety units based on metabolic transformations, i.e. a moiety model. The SAGA-optimize package, solving a boundary-value inverse problem through a combined simulated annealing and genetic algorithm, was developed for model optimization. Additional optimization methods from the Python scipy library are utilized as well. Several forms of the Akaike information criterion and Bayesian information criterion are provided for selecting between moiety models. Moiety models and associated isotopologue data are defined in a JSONized format.

By testing the moiety modeling framework on the timecourses of ^13^C isotopologue data for uridine diphosphate N-acetyl-D-glucosamine (UDP-GlcNAc) in human prostate cancer LnCaP-LN3 cells, we were able to confirm its robust performance in isotopologue deconvolution and moiety model selection.

**Conclusions:**

SAGA-optimize is a useful Python package for solving boundary-value inverse problems, and the moiety_modeling package is an easy-to-use tool for mass spectroscopy isotopologue profile deconvolution involving single and multiple isotope tracers. Both packages are freely available on GitHub and via the Python Package Index.

## Background

Recent work indicates that many human diseases involve metabolic reprogramming that disturbs normal physiology and causes serious tissue dysfunction [1]. Advances in analytical technologies, especially mass spectroscopy (MS) and nuclear magnetic resonance (NMR), have made metabolic analysis of human diseases a reality [2]. Stable isotope tracing is a powerful technique that enables the tracing of individual atoms through metabolic pathways. Stable isotope-resolved metabolomics (SIRM) uses advanced MS and NMR instrumentation to analyze the fate of stable isotopes traced from enriched precursors to metabolites, providing richer metabolomics datasets for metabolic flux analyses. NMR can measure isotopomer-specific metabolite data, but is typically limited by sensitivity. Often a single piece of NMR data only provides information on the presence of stable isotopes in just a part of a metabolite, which represents a partial isotopomer. In some cases, multiple partial isotopomer information can be interpreted in terms of a full isotopomer. MS can measure isotopologue-specific data; however, an isotopologue represents a set of mass-equivalent isotopomers. Comprehensive metabolic analysis often relies on MS metabolic datasets or a combination of MS and NMR metabolic datasets. Even though large amounts of metabolomics datasets have been generated recently, it is still a big challenge to acquire meaningful biological interpretation from MS raw data, especially for complex metabolites composed of multiple subunits or moieties.

To better interpret complex isotopologue profiles of large composite metabolites, both quantitative analysis as well as detailed modeling are required. Several methods have been developed for quantitative flux analysis of specified pathways based on the stable isotope incorporated data, like the elementary metabolite units (EMU) framework [3]. These methods rely heavily on well-curated metabolic networks to accomplish the metabolic flux analysis. However, models of cellular metabolism, even for human, are far from complete.

**Fig. 1:**
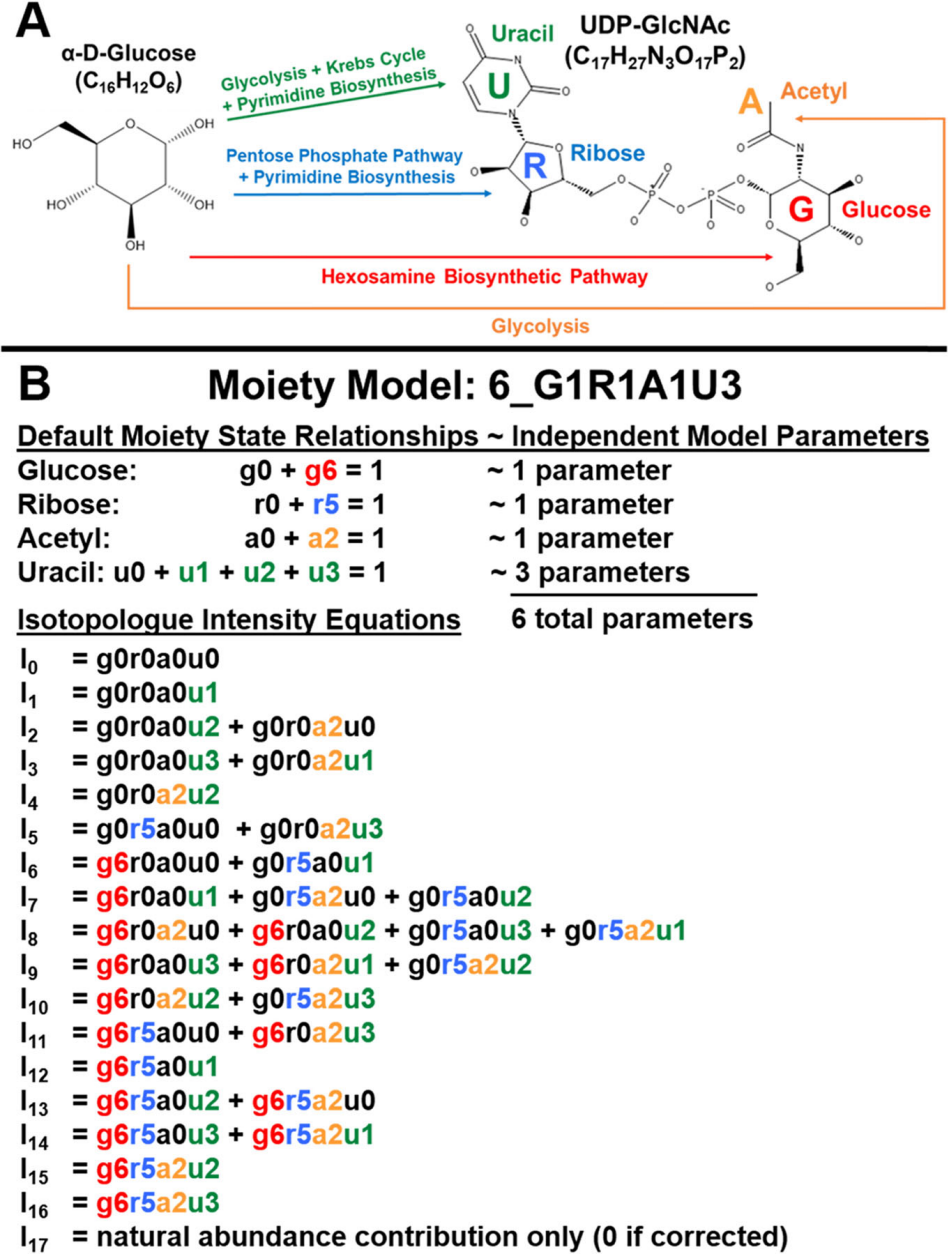
Example complex metabolite UDP-GlcNAc and associated expert-derived moiety model. **a** Major human metabolic pathways leading from glucose to the four moieties of UDP-GlcNAc. **b** The representative moiety model is based on the expected metabolic tracing from ^13^C-labeled glucose to UDP-GlcNAc, with the exception of one carbon in the uracil moiety that traces from carbon dioxide. The moiety states variables are identified by a lowercase moiety letter followed by a number representing the ^13^C isotope content. The moiety state variables (model parameters) are used to calculate specific components of the relative isotopologue intensity

To deconvolute the relative isotope incorporation fluxes of complex metabolites, first a plausible model of isotope incorporation should be built based on a relevant metabolic network, which is often incomplete. For example, the complex metabolite uridine diphosphose N-acetyl-D-glucosamine (UDP-GlcNAc), illustrated in Fig. 1a, has four distinct moieties in which ^13^C isotopes incorporate through a metabolic network from an isotope labeling source like ^13^C-labeled glucose. Based on the well-studied metabolic pathways that trace from glucose to UDP-GlcNAc in human metabolism, the expected (expert-derived) moiety model of ^13^C isotope incorporation from ^13^C-labeled glucose is illustrated in Fig. 1b, which includes ^13^C incorporation states for each moiety. For example, the g6 state represents the incorporation of ^13^C_6_ into the glucose moiety. Furthermore, the sum of moiety states for a given moiety is equal to 1. With this moiety model, a UDP-GlcNAc isotopologue profile can be deconvoluted into relative ^13^C isotope incorporation into each UDP-GlcNAc moiety: glucose, ribose, uracil, and acetyl. The deconvolution occurs by minimizing an objective function that compares calculated isotopologues based on moiety isotope incorporation (enrichment) state parameters from the model to the directly observed, experimentally-derived isotopologues. From a mathematics perspective, the minimization represents a highly non-linear inverse problem, since the experimental intensities are compared to calculated values from nonlinear equations that use model parameters being optimized (Fig. 1b). With a time-series of isotopologue profiles, relative isotope fluxes for each moiety can be derived and used for the interpretation of isotope flux through specific metabolic pathways associated with each moiety. However, when multiple models are plausible, development of a robust model selection method is essential for successful isotopologue deconvolution, especially for non-model organisms. This basic approach to isotopologue deconvolution was demonstrated in a prototype Perl program called GAIMS for the metabolite UDP-GlcNAc using a MS isotopologue profile derived from a prostate cancer cell line [4, 5]. This demonstration derived relative ^13^C isotope fluxes for several converging biosynthetic pathways of UDP-GlcNAc under non-steady-state conditions. This demonstration also inspired the development of MAIMS, a software tool for metabolic tracer analysis [6], which further validates the robustness of the moiety model deconvolution method. However, the MAIMS software handles only ^13^C single isotope tracer data and does not address model selection, which is crucial for addressing incomplete knowledge of cellular metabolic networks.

In addition, the simultaneous use of multiple stable isotopes in SIRM experiments can provide much more data than a single tracer. However, incorporation of multiple stable isotopes also complicates the analysis of metabolite isotopologue profiles, which limits most of the current isotope tracer experiments to a single tracer. The lack of data analysis tools greatly impedes the application of the multiple-labeled SIRM experiments. Therefore, we have developed a new moiety modeling framework for deconvoluting MS isotopologue profiles for both single and multiple-labeled SIRM MS datasets. This moiety modeling framework not only solves the non-linear deconvolution problem, but also facilitates selection of the optimal model describing the relative isotope fluxes for a specific metabolite(s) from a set of plausible models.

### Implementation

#### Overview of the moiety modeling framework

**Fig. 2:**
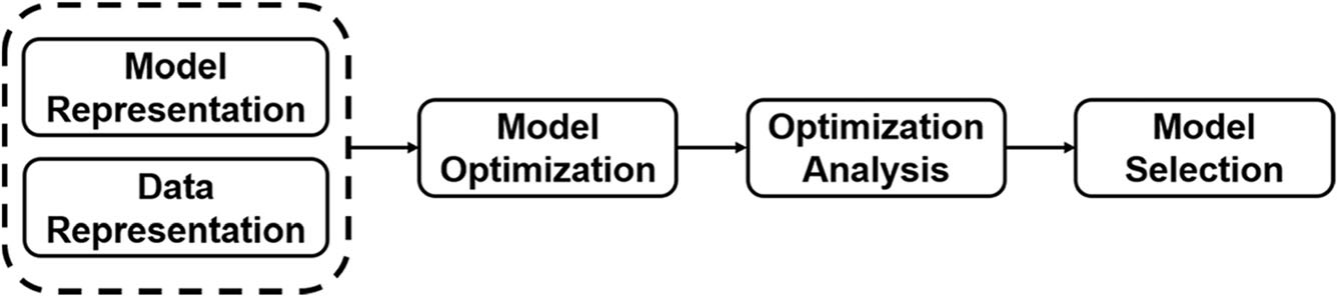
Workflow of the moiety modeling framework

**Fig. 3:**
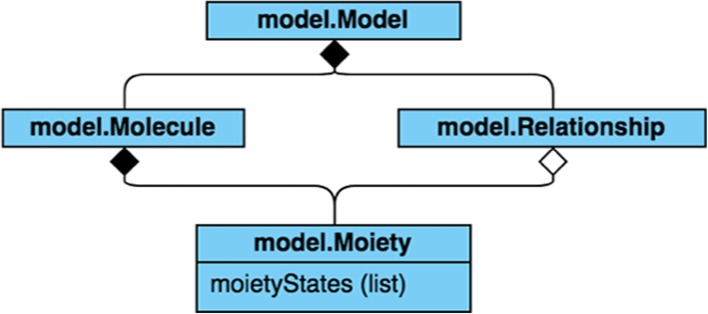
A unified modeling language (UML) class diagram of a Moiety Model

The workflow of the moiety modeling framework is composed of four major steps, model and data representation, model (parameter) optimization, analysis of optimization results, and model selection (Fig. 2). For the model and data representation step, the moiety_modeling package creates an internal representation of a moiety model from a given JSONized moiety model description (see Additional file 1). In this representation illustrated by a unified modeling language (UML) class diagram in Fig. 3, the package first dissembles a complex metabolite into a list of moieties, i.e. metabolic subunits. Each moiety may contain different number of labeling isotopes, representing the flow of isotope from the labeling source to the moiety. A moiety with a specific number of labeled isotopes is represented as an isotope enrichment state of the moiety (i.e. moiety state). As specified in the JSONized model description, non-default mathematical relationships may exist between moiety states, even from different moieties and/or molecules. Molecules, their moieties, the possible moiety states, and relationships between moiety states work together to represent a particular moiety model, and the proportion for each possible moiety state is an optimizable parameter of the model. Each mass spectrum’s worth of isotopologue data is represented as a separate dataset, which holds the set of isotopologues associated with each molecule. Typically, multiple mass spectra are included. Often each mass spectrum represents a single time point in a time series experiment.

The next major step, moiety model (parameter) optimization, involves deriving an optimal set of model parameters, i.e. moiety state fractional abundances (*moiety* _ *state*_*j*, *i*_ for moiety j and state i) that are used to calculate relative isotopologue abundances (*I*_*x,calc*_ from Eq. 1) that best match experimental isotopologue profiles (*I*_*x,obs*_) as compared by an objective function (see Table 1). In Eq. 1, ic_a_ is a component of the isotopologue intensity with an isotope content x. Figure 1b lists these isotopologue components for each isotopologue based on the expert-derived moiety model.

**Table 1:** Different forms of objective function

Loss function	Equation
Absolute difference	Σ|I_*x,obs*_ – I_*x,calc*_|
Log difference	Σ|log (I_*x,obs*_) – log (I_*x,calc*_)|
Square difference	Σ (I_*x,obs*_ – I_*x,calc*_)^2^

1 $$ {I}_{x, calc}=\sum \limits_{ic_a\in {IC}_x}{ic}_a;{IC}_x=\left\{{ic}_v| isotope\_ content\left({ic}_v\right)=x\right\};{ic}_v={\prod}_j moiety\_{state}_{j,{v}_j} $$

The moiety_modeling package implements several optimization methods, including a combined simulated annealing and genetic algorithm (SAGA) based on the ‘Genetic Algorithm for Isotopologues in Metabolic Systems’ (GAIMS) Perl implementation [4, 5], a truncated Newton algorithm (TNC) [7], a SLSQP algorithm using Sequential Least Squares Programming [8], and a L-BFGS-B algorithm [9]. For the latter three algorithms ‘TNC’, ‘SLSQP’, and ‘L-BFGS-B’, the moiety_modeling package uses the implementation from the scipy.optimize Python module. In addition, we have the option to optimize the datasets together or separately.

The third major step involves the analysis of the results from the model optimization. The moiety_modeling package provides facilities for generating summative statistics and graphical visualizations for a set of optimizations performed on one or more moiety models. The final major step, model selection, tries to find the model that best fits the experimental isotopologue profiles from a set of provided moiety models that have been optimized in step two. Several forms of the Akaike information criterion (AIC) [10] and Bayesian information criterion (BIC) [11] are used as the estimator of the relative quality of moiety models for the set of isotopologue data.

#### The moiety_modeling python package implementation

**Fig. 4:**
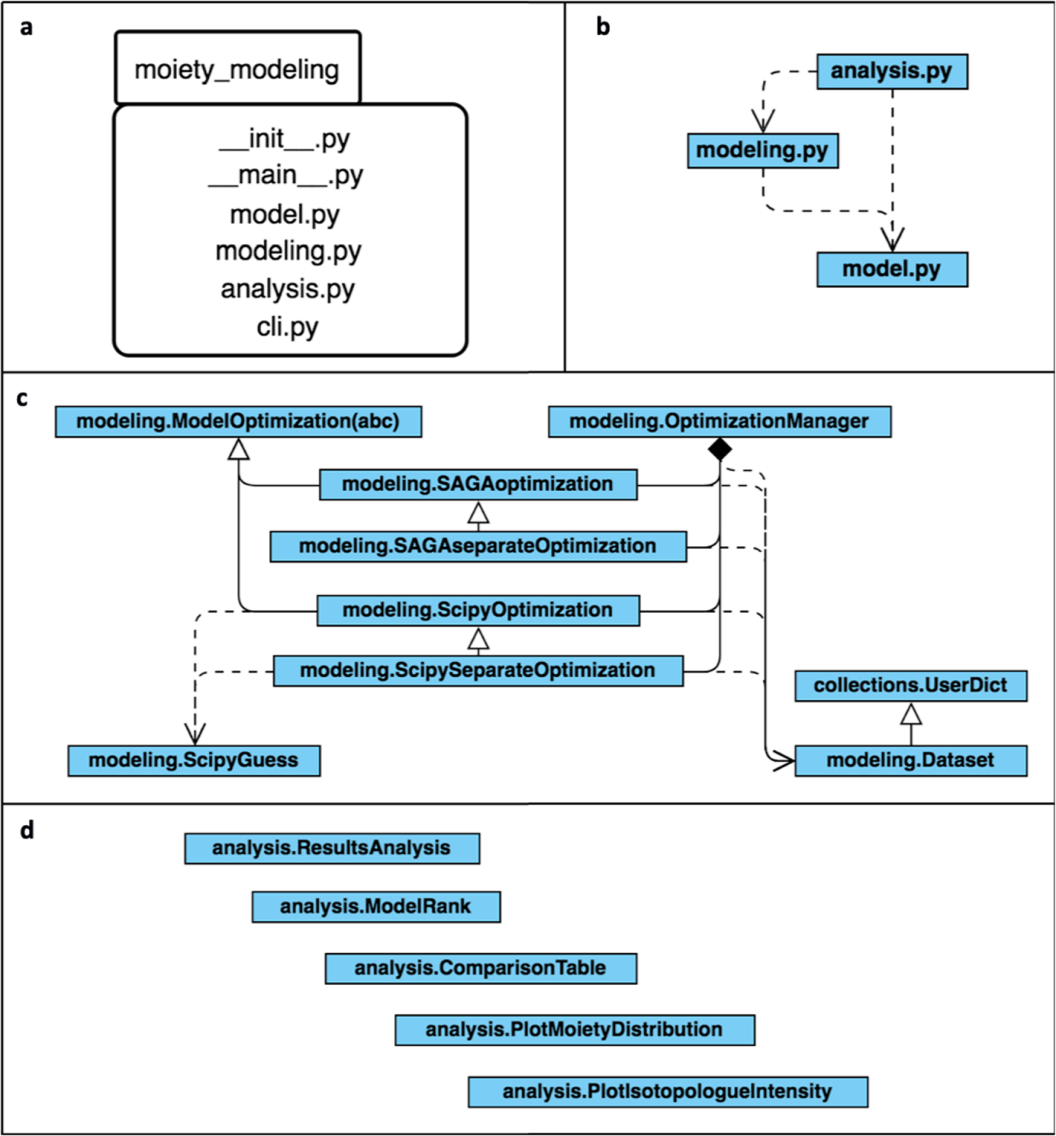
Organization of the moiety_modeling package represented with UML diagrams: **a** UML package diagram of the moiety_modeling Python library; **b** Subpackage dependencies diagram; **c** UML class diagram of the ‘modeling.py’ module with dependency relationships; **d** UML class diagram of the ‘analysis.py’ module, which contains a set of classes with no relationships

As shown in Fig. 4, the moiety_modeling Python package consists of several modules: ‘model.py’, ‘modeling.py’, ‘analysis.py’, and ‘cli.py’. The ‘model.py’ module contains class definitions for the basic elements in the moiety model. It is composed of ‘Moiety’, ‘Relationship’, ‘Molecule’ and ‘Model’ classes. The ‘Moiety’ object represents a specific moiety, the labeling isotopes present in the moiety, and their corresponding states within the moiety. The ‘Relationship’ class describes the non-default mathematical dependencies between moiety states, where the default dependency for a given moiety is that the sum of its states is equal to 1 (see Fig. 1b for example default relationships). A ‘Molecule’ object represents an individual metabolite made up of a list of ‘Moiety’ objects. The ‘Model’ class simulates the flow of isotope from labeling sources into each moiety of specific metabolites, which is initialized by lists of ‘Moiety’ objects, ‘Molecule’ objects, and ‘Relationship’ objects. A moiety model is generated and stored in a JSONized representation using the jsonpickle Python package [12]. This JSONized representation (see Additional file 2), stored in a file, is then used as the input file for later model optimizations. The ‘modeling.py’ module is responsible for model optimization. It is composed of the ‘Dataset’ class, several model optimization classes, and the ‘OptimizationManager’ class. The ‘Dataset’ class organizes a single MS isotopologue profile dataset into a dictionary-based data structure. ‘Dataset’ objects are stored in a JSONized representation (see Additional file 3) and used as the input for later model optimizations. Currently, no relationship between Dataset objects like a time-dependence is captured. In the abstract ModelOptimization class, we included several different objective functions (see Table 1). In addition, there are four specific model optimization classes in the ‘modeling’ module that utilize different optimization methods and approaches for combining datasets. The ‘SAGAoptimization’ and ‘SAGAseparateOptimization’ classes use the SAGA-optimize Python package described in the next section for either combined optimization of model parameters across all datasets or separate optimizations of model parameters for each dataset. ‘ScipyOptimization’ and ‘ScipySeparateOptimization’ classes make use of optimization methods (‘TNC’, ‘SLSQP’, and ‘L-BFGS-B’) in the scipy.optimize module to conduct optimizations in either a combined or separate manner. The ‘OptimizationManager’ class is responsible for the management of the optimization process based on the input optimization parameters. The results for a model optimization are stored in a JSONized representation (see Additional file 4) for further analysis. A text file is used to store the filepaths to all of the optimized models with certain optimization parameters. The filepath file is then used as the input for the ‘analysis.py’ module. The ‘analysis.py’ module has five classes: ‘ResultsAnalysis’, ‘ModelRank’, ‘ComparisonTable’, ‘PlotMoietyDistribution’ and ‘PlotIsotopologueIntensity’. The ‘ResultsAnalysis’ class is responsible for generating standard statistics from the results for a set of optimizations for a given model. The mean, standard deviation, minimum, and maximum value of each model parameter are calculated from a set of model optimizations performed on the same model. The calculated isotopologue intensities and their statistics based on the sets of optimized parameters are also generated. Furthermore, several quality estimators of each model, including different forms of the ‘AIC’ (Table 2), are computed for model selection. The AIC tends to select the model that has too many parameters when the sample size is small, leading to overfitting. The sample size corrected AIC (AICc) was developed to address this overfitting problem [13]. The Bayesian information criterion (BIC) is another commonly used criterion for model selection [14]. The ‘ResultsAnalysis’ objects with results for each model are stored in a JSONize representation (see Additional file 5) for further analysis, along with a text report for readability. Also, an analysis filepath file containing the filepaths to the analysis JSON files of all models with the same optimization parameters is created. Next, the ‘ModelRank’ class object uses this analysis filepath file to compare and select the model that best reflects the observed isotopologue profile. The ‘ComparisonTable’ class compares the model selection results with different optimization parameters. The ‘PlotMoietyDistribution’ class and ‘PlotIsotopologueIntensity’ class are responsible for the visualization of the optimization results for a set of optimizations performed on a single model. The ‘cli.py’ module provides the command-line interface to perform model optimization, model optimization analysis, and model selection, which is implemented with the ‘docopt’ Python library [15].

**Table 2:** Different forms of a model selection estimator

Selection Criterion	Equation
Akaike Information Criterion (AIC)	2k + nln(RSS/n)
Sample size corrected AIC (AICc)	AIC + (2*k*^2^ + 2k)/(n − k − 1)
Bayesian Information Criterion (BIC)	nln(RSS/n) + kln(n)

k is the number of parameters

n is the number of data points

RSS is the residual sum of squares: RSS = $\sum \limits_{i=1}^n{\left({I}_{obs}-{I}_{calc}\right)}^2$

#### SAGA-optimize python package implementation

**Fig. 5:**
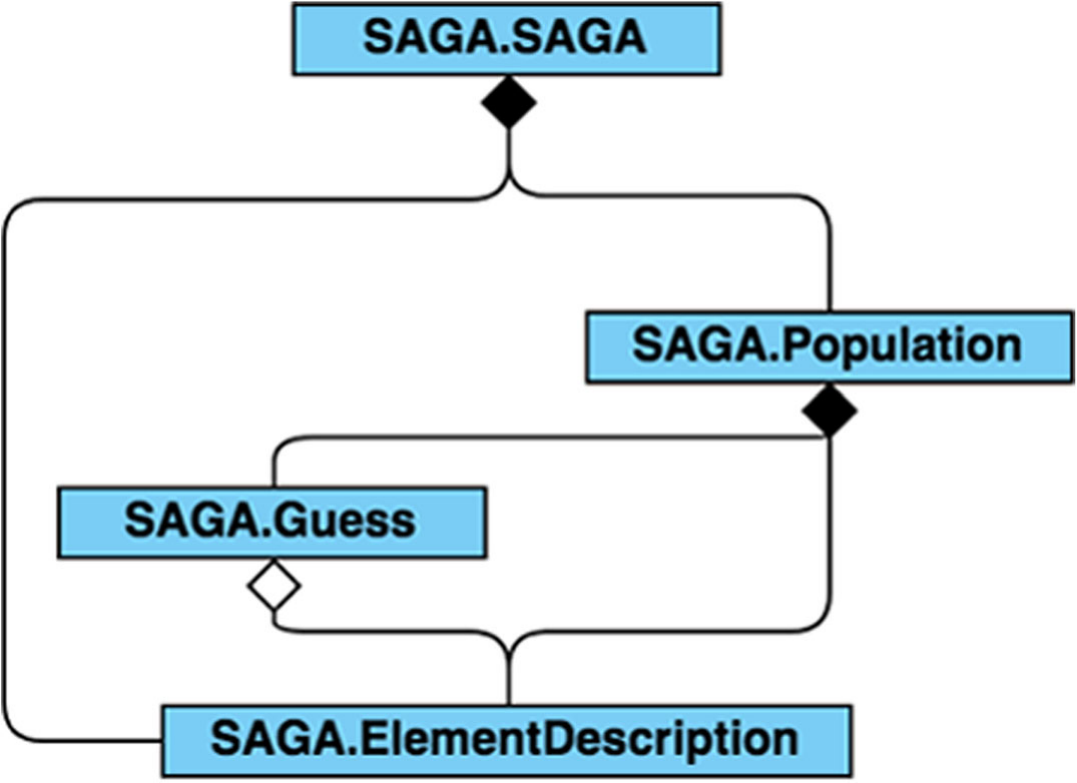
‘SAGA-optimize’ package represented with a UML class diagram with dependencies

The SAGA-optimize Python package is a novel type of combined simulated annealing and genetic algorithm [4] used to find the optimal solutions to a set of parameters based on the minimization of a given energy (objective) function calculated using the set of parameters. In this context, the energy function represents a comparison of calculated and experimentally-observed isotopologue relative intensities, with the calculated intensities based on the moiety model parameters being optimized. As shown in Fig. 5, it is composed of ‘ElementDescription’, ‘Guess’, ‘Population’ and ‘SAGA’ classes. An ‘ElementDescription’ object describes an individual parameter of the moiety model. In the expert derived moiety model (Fig. 1b), the g6 model parameter would be represented by a single ‘ElementDescription’ object. The ‘ElementDescription’ object is bound by a range and several mutation methods are available to change the value of the ‘ElementDescription’ object. A ‘Guess’ object contains lists of all the parameters (‘ElementDescription’ objects) and their corresponding values for a particular moiety model. In addition, it also stores the energy calculated based on this set of parameters. A ‘Population’ object contains information of a list of ‘ElementDescription’ objects, a list of ‘Guess’ objects, the range of each ‘ElementDescription’ among all the ‘Guess’ objects, the highest and lowest energy for the list of ‘Guess’ objects, and the best ‘Guess’ object. The ‘ElementDescription’, ‘Guess’ and ‘Population’ classes are the building blocks of the ‘SAGA’ class, which is the main class that provides the interface for optimization. Furthermore, several distinct crossover functions are available for creating new Guess objects from the cross-over of two other Guess objects.

## Results

### The package interface

The moiety_modeling package can be used in two main ways: (i) as a library within Python scripts for accessing and manipulating moiety models and isotopologue datasets stored in JSON files, or (ii) as a command-line tool to perform model optimization, model analysis, and model selection.

To use the moiety_modeling package as a library within Python scripts, it should be imported with a Python program or an interactive interpreter interface. Next, ‘Moiety’, ‘Relationship’ and ‘Molecule’ objects can be created to construct a moiety model. ‘Dataset’ objects are also built with the moiety_modeling package. Table 3 summarizes common patterns for using moiety_modeling package as a library in construction of a moiety model and related datasets.

**Table 3:** Common creation patterns for the moiety_modeling library

Entity	Example
Moiety	glucose = moiety_modeling.Moiety(‘glucose’, {‘13C’: 6}, isotopeStates = {‘13C’: [1, 3, 5]}, nickname = ‘g’) acetyl = moiety_modeling.Moiety(‘acetyl’, {‘13C’: 2}, isotopeStates = {‘13C’: [0, 1, 2]}, nickname = ‘a’) uracil = moiety_modeling.Moiety(‘uracil’, {‘13C’: 4}, isotopeStates = {‘13C’: [1, 2, 4]}, nickname = ‘u’) ribose = moiety_modeling.Moiety(‘ribose’, {‘13C’: 5}, isotopeStates = {‘13C’: [0, 3, 5]}, nickname = ‘r’)
Relationship	relationship = moiety_modeling.Relationship (glucose, ‘13C0’, acetyl, ‘13C2’, ‘*’, 2)
Molecule	UDP-GlcNAc = moiety_modeling.Molecule(‘UDP-GlcNAc’, [glucose, uracil, acetyl, ribose])
Model	model1 = moiety_modeling.Model(‘model1’, [glucose, uracil, acetyl, ribose], [UDP_GlcNAc], [relationship])
Dataset	dataset = moiety_modeling.Dataset(‘12 h’, ‘UDP_GlcNAc’: [{‘labelingIsotopes’:‘13C_0’, ‘height’: 0.0175, ‘heightSE’: 0}, {‘labelingIsotopes’:‘13C_1’, ‘height’: 0.0075, ‘heightSE’: 0}, …])

The moiety_modeling package also provides a simple command-line interface to perform model optimization, selection, and visualization. Additional file 6 shows version 1.0 of the command-line interface, and Table 4 summarizes common pattern for using moiety_modeling as a command-line tool. The common patterns for using SAGA-optimize as a library are shown in Additional file 7.

**Table 4:** Common patters for using the moiety_modeling as a command-line tool

Command	Description	Example
modeling	Perform model optimization	% python3 –m moiety_modeling modeling --models = models.json --datasets = dataset.json --optimizations = optimization_settings.json
analyze	Analyze the optimization results	% python3 –m moiety_modeling analyze optimizations --a optimizationPaths.txt
plot	Plot the distribution of calculated moiety modeling parameters.	% python3 –m moiety_modeling plot moiety analysisResults.json

### Dataset and model

We used the timecourse (34 h, 48 h, and 72 h) of ^13^C isotopologue data for UDP-GlcNAc generated from [U-^13^C]-glucose in human prostate cancer LnCaP-LN3 cells to evaluate the robustness of the moiety modeling framework. An expert-derived moiety model of UDP-GlcNAc (6_G1R1A1U3) was created based on known human biochemical pathways (Fig. 1a) and corroborated by NMR data. Also, 40 hypothetical moiety models of the isotopic flow into UDP-GlcNAc were crafted as simple perturbations of the original expert-derived model. These perturbations include the inclusion of different and/or additional moiety states and non-default moiety state relationships (e.g. g6 = r5). For example, model 7_G2R1A1U3_g5 includes an extra ^13^C_5_ g5 glucose moiety state for a total of 7 independent model parameters, 2 for glucose, 1 for ribose, 1 for acetyl, and 3 for uracil. We tested whether the expert-derived moiety model could be selected from all the other models.

### Model optimization and selection

The incorporation of ^13^C from [U-^13^C]-glucose into UDP-GlcNAc leads to a total of 17 isotopologues plus one due to ^13^C natural abundance from carbon dioxide (I_0_, …, I_17_). We applied the moiety modeling framework to the observed UDP-GlcNAc isotopologue data with each built model to test whether the expert-derived moiety model could be selected above the other models. We used the SAGA optimization method with a log difference objective function (see Table 1). The optimization was repeated 100 times for each model. These analyses were performed on a desktop computer with i7-6850K CPU (6 core with HT), 64GB RAM and 512GB SSD. On this hardware, the analyses for all 40 models took roughly 3 h of total execution time. The results are listed in Table 5. From these results, we can see that the expert-derived moiety model can be selected successfully among all the moiety models using the AICc (see Table 2), which demonstrates the robustness of the moiety modeling framework. Model selection criteria like the AICc help to address model overfitting; however, the use of a log difference objective function with multiple time points of data in the form of separate sets of observed isotopologues makes the model selection very robust against most of the model overfitting [4, 5].

**Table 5:** Model selection results of UDP-GlcNAc isotopologue data

Model^a^	Estimator (AICc)
6_G1R1A1U3 (expert-derived model)	− 229.2918
6_G1R1A1U3_r4	− 227.5208
6_G1R1A1U3_u4	− 225.0006
6_G0R2A1U3_g3r2r3_g6r5	− 223.1633
6_G1R1A1U3_g5	− 215.9565
7_G1R2A1U3_r1	− 212.4727
7_G2R1A1U3_g1	−212.1217
7_G1R2A1U3_r3	−210.9640
7_G1R1A2U3	−210.0952
7_G2R1A1U3_g5	−208.1346
7_G1R2A1U3_g3r2r3	− 207.6523
7_G1R2A1U3_r2	−207.4187
7_G2R1A1U3_g4	− 206.6430
7_G2R1A1U3_g2	−206.5609
7_G0R2A2U3_g3r2r3_g6r5	− 205.0569
7_G2R1A1U3_g3	− 204.8797
7_G0R3A1U3_g3r2r3_g6r5_g5r4	−204.2729
7_G1R1A1U4	− 203.3710
7_G1R2A1U3_r4	− 202.6782
6_G1R1A1U3_a1	−199.5560
8_G2R1A2U3_g1	− 195.9713
7_G1R1A1U3C1	− 195.5788
8_G1R2A2U3_r1	−195.4893
7_G0R3A1U3_g3r2r3_g6r5_r4	−192.4980
8_G1R2A2U3_r2r3	−187.3342
8_G1R2A2U3_r3	−186.8810
8_G2R1A2U3_g5	−186.2693
8_G1R2A2U3_r2	−186.2562
8_G2R1A2U3_g2	− 185.6112
8_G2R1A2U3_g4	− 184.9444
8_G1R2A2U3_g3r2r3	−184.2929
8_G1R2A2U3_g3r2r3_g6r5_g5	− 183.2154
8_G2R1A2U3_g3	−183.1467
8_G1R2A2U3_r4	− 182.1334
8_G1R1A2U3C1	− 177.5013
9_G2R2A2U3_r2r3_g1	− 170.3323
9_G2R2A2U3_r2r3_g2	− 161.5770
9_G2R2A2U3_r2r3_g3	− 160.7823
9_G2R2A2U3_r2r3_g6r5_g3_g5	−160.6917
9_G2R2A2U3_r2r3_g4	−160.4500
9_G2R2A2U3_r2r3_g5	− 158.8733

Optimization settings: method = ‘SAGA’, SAGA_parameters = {‘stepNumber’: 100000, ‘temperatureStepSize’: 100, ‘alpha’: 1, ‘crossoverRate’: 0.05, ‘mutationRate’: 3, ‘populationSize’: 20, ‘startTemperature’: 0.5}, repetition = 100, split, objective function = log difference

^a^The first number in the model name is the total number of free model parameters followed by the number of free parameters for each moiety and perturbations from the expert-derived model

We also compared the optimization results generated by the moiety-modeling package to results generated by GAIMS (see Additional files 9, 10, 11). For this comparison, an absolute difference objective function was used with the moiety-modeling package to match the objective function available in the GAIMS software. Also, there are some small differences in the implementation of optimization method between the two software packages. The SAGA-optimize package implements a true simulated annealing, while GAIMS implements a modified annealing with steepest decent qualities. Also, both optimization methods are stochastic as demonstrated by replicate moiety-modeling analyses shown in Additional file 12. Therefore, the results are not identical; however, they are reasonably comparable. But neither method is able to select the expert-derived model with an AICc model selection method, due to issues of overfitting with the absolute difference objective function.

### Generation of simulated single-tracer and multi-tracer datasets

In addition, we generated simulated single tracer and multi-tracer datasets to test, compare, and evaluate multi-tracer optimization functionality. First, we created a set of rounded moiety state values for the single-tracer expert derived model roughly based on the optimized model state values derived from the experimental UDP-GlcNAc 48 h dataset (Table 6).

**Table 6:** Single-tracer ^13^C moiety states and values for UDP-GlcNAc biosynthesis

Moiety states	Moiety value	Moiety states	Moiety value
glucose[13C_0]	0.1	ribose[13C_5]	0.9
glucose[13C_6]	0.9	uracil[13C_0]	0.2
acetyl[13C_0]	0.7	uracil[13C_1]	0.2
acetyl[13C_2]	0.3	uracil[13C_2]	0.5
ribose[13C_0]	0.1	uracil[13C_3]	0.1

We then used ^13^C and ^18^O labeled glucose (^13^C_6_H_12_^18^O_6_) as a hypothetical isotope labeling source for UDP-GlcNAc biosynthesis. Following the expert derived model and with the aid of atom-mapping information of relevant human biochemical reactions from MetaCyc [15], we traced the incorporation of oxygen and carbon atoms from glucose to each moiety to derived a multi-tracer model. For glucose, acetyl and ribose, oxygen atoms incorporated into the moiety with their directly bonded carbon atom. However, during the biosynthesis of uracil, some ^18^O-^13^C bonds are sometimes broken, creating a more varied set of moiety states. Next, we derived rounded multi-tracer moiety state values that are equivalent to the rounded single-tracer values (Table 7).

**Table 7:** Multi-tracer ^13^C/^18^O moiety states and values for UDP-GlcNAc biosynthesis

Moiety states	Moiety value	Moiety states	Moiety value
glucose[13C_0.18O_0]	0.1	uracil[13C_0.18O_0]	0.2
glucose[13C_6.18O_5]	0.9	uracil[13C_1.18O_0]	0.2
acetyl[13C_0.18O_0]	0.7	uracil[13C_2.18O_0]	0.25
acetyl[13C_2.18O_1]	0.3	uracil[13C_2.18O_1]	0.25
ribose[13C_0.18O_0]	0.1	uracil[13C_3.18O_0]	0.05
ribose[13C_5.18O_4]	0.9	uracil[13C_3.18O_1]	0.05

Next, we generated the base single-tracer and multi-tracer simulated datasets by calculating the set of relative isotopologue intensity values using Eq. 1 with the respective moiety state values. Finally, we created simulated datasets with added normally distributed error that is subsequently thresholded to zero based on a minimum hypothetical detection limit (0.005) and then renormalized to a sum of 1. We generated three sets of 100 simulated datasets for both single and multi-tracer models by adding error from a normal distribution with increasing standard deviations of 0.001, 0.01 and 0.1. We then estimated the effects of error propagation by calculating the average sum of isotopologues across 100 simulated datasets after error addition and thresholding, but before renormalization (Table 8).

**Table 8:** Average sum of simulated isotopologues before renormalization

σ of Added Error	Average Sum of Isotopologues	
	Single-tracer	Multi-tracer
0.1	1.50	9.97
0.01	1.02	1.73
0.001	0.99	0.98

**Fig. 6:**
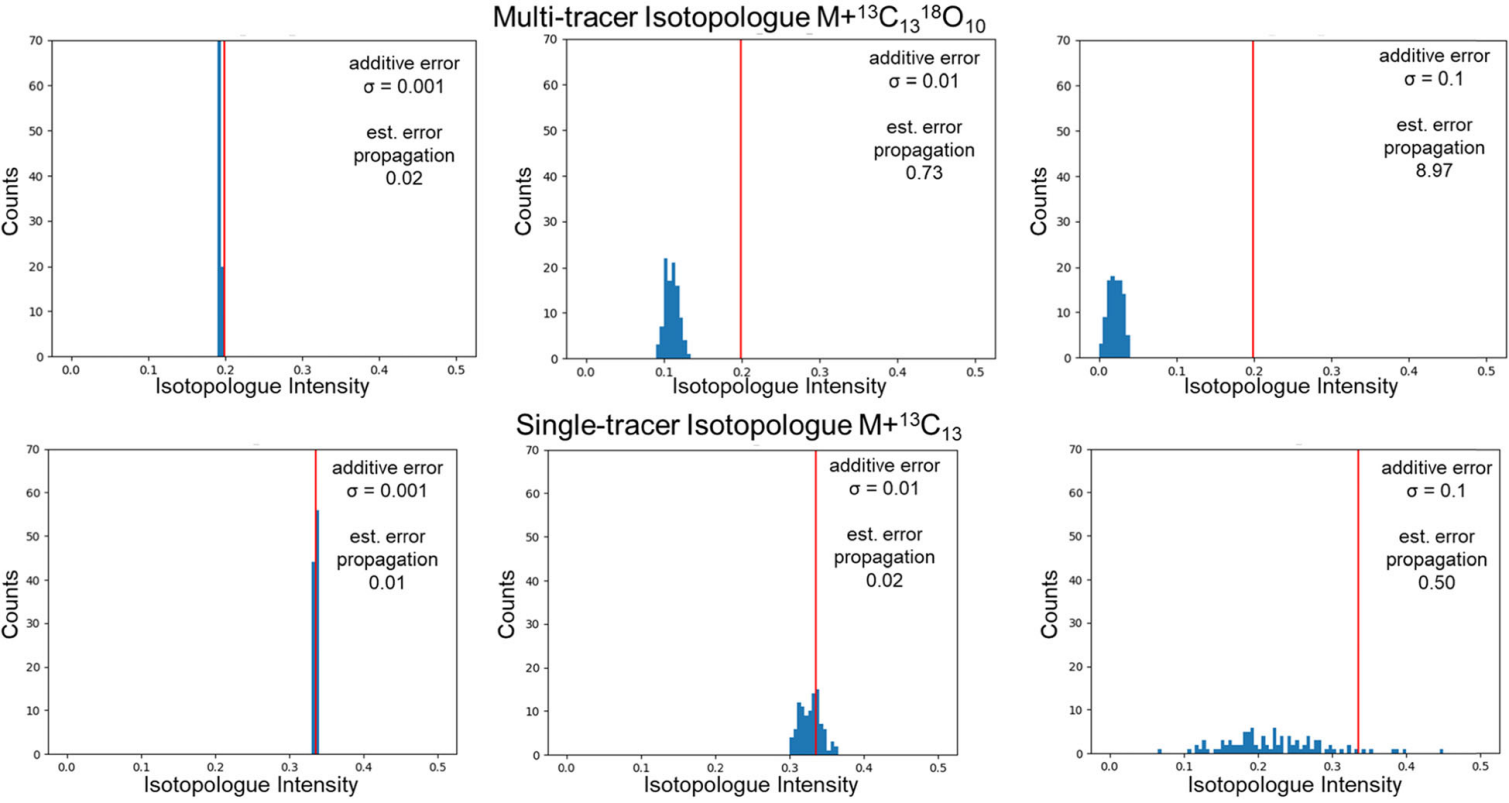
Histograms of simulated intensities for the largest representative isotopologue

Based on this calculation, the single-tracer datasets and the multi-tracer datasets have comparable levels of propagated error when normal error with a 0.001σ is added. However, this quickly deviates with larger amounts of additive error as shown by single-tracer datasets with a 0.1σ added normal error having slightly less propagated error than the multi-tracer datasets with a 0.01σ added normal error. The multi-tracer datasets with a σ = 0.1 added normal error are practically useless due to the level of propagated error being roughly nine (i.e. 9.97–1.00 = 8.97 ≈ 9) times the original signal on average. Using histograms of simulated intensities for the largest respective isotopologue in both the single-tracer and multi-tracer simulated datasets, Fig. 6 illustrates these error propagation effects due to thresholding and renormalization. It is clear from this figure the loss of intensity information in the multi-tracer simulated dataset with σ = 0.1 added normal error.

### Model optimization of simulated multi-tracer and single-tracer datasets and comparison of results

**Fig. 7:**
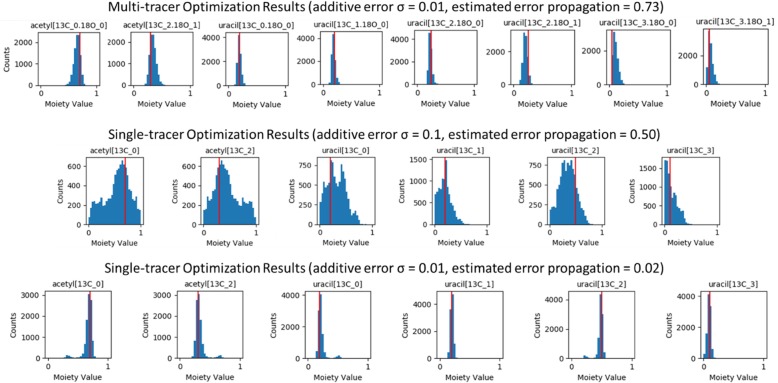
Histograms of the acetyl and uracil optimized moiety state values derived from simulated datasets

For each simulated dataset consisting of a single time point, the respective model was optimized 100 times (i.e. in 100 separate repetitions), each using 5000 steps of SAGA with an absolute objective function. This generated 10,000 separate optimizations for each set of simulated datasets at a given added level of error. Using histograms, Fig. 7 visualizes the distribution for the acetyl and uracil moiety state values for the multi-tracer dataset with 0.01σ added normal error and for the single-tracer datasets with σ = 0.1 and σ = 0.01 added normal error. The full set of histograms are in Additional file 13 for the multi-tracer results and Additional file 14 for the single tracer results. When comparing multi-tracer and single-tracer experiments with equivalent added normal error (σ = 0.01), the propagated error leads to wider variances in the multi-tracer moiety state values and some additional skewness of their distributions. However, some of the single-tracer moiety state value distributions are bimodal. When comparing multi-tracer and single-tracer experiments with comparable propagated error levels, the multimodality in the single-tracer distributions become very pronounced, especially in the acetyl moiety states.

## Discussion

### Advantage of JSONized representation for MS isotopologue data and analysis results

JavaScript object notation (JSON) [16] is an open-standard file format using human-readable text to collect data in pair-value and array structures, widely used by different programming language. Complex Python objects, like ‘Moiety’ and ‘Molecule’ objects mentioned above, can be serialized to JSON format with the jsonpickle Python library. The moiety model and dataset constructed with moiety_modeling package as well as optimization parameters are the input files for the moiety modeling, all of which are saved in JSON format using jsonpickle (see Additional files 2, 3, and 8). The use of JSON format makes the moiety modeling framework easily accessible to other programming languages and naturally extendible. In addition, the optimization and analysis results are also stored in a JSON file (see Additional files 4 and 5).

### Advantages and limitations of the SAGA-optimize and moiety-modeling packages

The SAGA-optimize package provides certain advantages to the model optimization versus the other optimization methods from scipy and even a similar implementation in GAIMS. The level and steepness of optimization can be precisely tuned with the specification of the annealing length and schedule. Also, this novel implementation of a combined simulated annealing and genetic algorithm incorporates the annealing processing directly into the mutation step itself, attenuating the level of mutation as the annealing temperature drops. The moiety-modeling package provides a range of objective functions and can split each independent set of isotopologues into individual moiety model optimizations, which neither the GAIMS nor MAIMS packages can do. Moreover, both the SAGA-optimize and moiety-modeling packages have multiprocessing facilities that enable an efficient utilization of all CPU cores. As demonstrated in the Table 5 results, the combination of advantages allows the moiety-modeling package to optimize and accurately select the expert-derived model in roughly one tenth of the execution time of the original GAIMS package, i.e. with 100,000 steps of optimization in moiety-modeling versus 1,000,000 steps in GAIMS. Also, both the SAGA-optimize and moiety-modeling packages contain over 2200 lines of code implemented in major version 3 of the Python language with a fully object-oriented design and Pythonic style. Every module, class, method, and function have documentation strings (docstrings) written in the reStructuredText markup language. Variables, data members, methods, functions, and classes have descriptive names as demonstrated in Figs. 3, 4, and 5. Documentation is automatically generated using the Sphinx Python Document Generator and made available on ReadTheDocs. This documentation includes a user guide, installation instructions, tutorial, and application programming interface (API) reference. Both packages are available on GitHub, utilize Travis CI for continuous integration, and are distributed via the Python Package Index. Code coverage from unit testing is above 65% for moiety-modeling and above 73% for SAGA-optimize. These packages enable researchers to perform moiety model isotopologue deconvolution using JSON representations of moiety models, datasets, and optimization method selection and settings provided by the user. At this time, the moiety-modeling package has no facilities for automatic moiety model generation.

### Difficulty in generating simulated datasets and comparing multi-tracer to single-tracer moiety modeling results

The generation of realistic simulated biophysical datasets is always a non-trivial task [16]. Even the addition of normal additive error can create non-intuitive propagation of error, especially through inverse problems [17]. This is illustrated in Table 8 and Fig. 6, where thresholding creates a positive bias in accumulated error and the renormalization creates a proportional-like error component from this positive accumulated error. The thresholding is required to keep the simulated data within the physical boundaries of the analytical detection, i.e. all non-negative values. The renormalization keeps the simulated data within mathematical boundaries, i.e. the sum of the isotopologue values is equal to 1. Neither step can be avoided with the inclusion of normal additive error. This created error propagation problem is quite dramatic for the simulated multi-tracer datasets, because there are 324 possible isotopologues in the multi-tracer datasets as compared to only 18 isotopologues in the single-tracer datasets. This problem simply increases in magnitude with the number of isotopologues present in a dataset. With a σ = 0.1 added normal error, the isotopologue intensity information is effectively lost for the multi-tracer datasets (see Fig. 6) and these datasets become effectively unusable (see Additional file 13). However, the lower additive error datasets are usable and illustrate the power of multi-tracer datasets to reduce multimodality in optimized moiety state values as compared to the single-tracer datasets.

## Conclusions

Here, we present a moiety modeling framework for the deconvolution of metabolite isotopologue profiles using moiety models along with the analysis and selection of the best moiety model(s) based on the experimental data. This framework can analyze datasets involving single and multiple isotope tracers as demonstrated on simulated datasets for multiple tracer models and both simulated and experimental datasets on single tracer models. With a ^13^C-labeled UDP-GlcNAc isotopologue dataset, we further demonstrate the robust performance of the moiety modeling framework for model selection on real experimental datasets. The selection of correct moiety models is required for generating deconvolution results that can be accurately interpreted in terms of relative metabolic flux. Furthermore, the JSON formats of moiety model, isotopologue data, and optimization results facilitate the inclusion of these tools in data analysis pipelines. Future work will explore the data quality requirements of model selection and validation of multiple isotope tracing model optimization and selection.

## Availability and requirements

**Project name:** moiety_modeling.

**Pipeline Installation manual:** https://moiety-modeling.readthedocs.io/en/latest/

**Operating system:** Linux.

**Programming language:** Python 3.5+.

**Other requirements:** jsonpickle, matplotlib, docopt, scipy, numpy.

**License:** BSD

## Supplementary information

**Additional file 1.** JSONized description of moiety model components.

**Additional file 2.** Moiety model description.

**Additional file 3.** Dataset for moiety modeling.

**Additional file 4.** Optimization results.

**Additional file 5.** Analysis results.

**Additional file 6.** The moiety_modeling package command line interface.

**Additional file 7.** Common patterns for using ‘SAGA’ module as a library.

**Additional file 8.** Optimization parameters.

**Additional file 9.** Model rank comparison between moiety_modeling and GAIMS.

**Additional file 10.** Comparison of optimized model parameters between moiety_modeing and GAIMS (Graph).

**Additional file 11.** Comparison of optimized model parameters between moiety_modeing and GAIMS (Table).

**Additional file 12.** Comparison of model rank between different repetitions.

**Additional file 13.** Multi-tracer optimization results for simulated datasets.

**Additional file 14.** Single-tracer optimization results for simulated datasets.

## Data Availability

The moiety_modeling and SAGA-optimize packages are available on: GitHub - https://github.com/MoseleyBioinformaticsLab/moiety_modeling, https://github.com/MoseleyBioinformaticsLab/SAGA_optimize . PyPI - https://pypi.org/project/moiety-modeling/, https://pypi.org/project/SAGA-optimize/ . Project documentation is available online at ReadTheDocs: https://moiety-modeling.readthedocs.io/en/latest/, https://saga-optimize.readthedocs.io/en/latest/ . All the results analyzed in this manuscript are available on figshare: https://figshare.com/articles/moiety_modeling_framework/7886135 .

## Abbreviations

AIC: Akaike information criterion
BIC: Bayesian information criterion
GAIMS: Genetic Algorithm for Isotopologues in Metabolic Systems
JSON: JavaScript Object Notation
MS: mass spectroscopy
NMR: nuclear magnetic resonance
SAGA: simulated annealing and genetic algorithm
SIRM: stable isotope-resolved metabolomics
UDP-GlcNAc: UDP-N-acetyl-D-glucosamine
UML: unified Modeling Language
