# Supplementary material for: Moiety modeling framework for deriving moiety abundances from mass spectrometry measured isotopologues
Source: BMC Bioinformatics. 2019 Oct 28;20:524. doi: 10.1186/s12859-019-3096-7 (PMC6816163; doi:10.1186/s12859-019-3096-7)
Supplement: Supplementary file 1 — Additional file 1. JSONized description of moiety model components. [file 12859_2019_3096_MOESM1_ESM.docx]

| **Moiety**  {  “name”: “ribose”,  “nickname”: “r”,  “ranking”: 1,  “maxIsotopeNum”: {“13C”: 5},  “isotopeStates”: {“13C”: [0, 2, 3, 5] },  “states”: [ “13C0”, “13C2”, “13C3”, “13C5” ] ,  “py/object”: “moiety_modeling.model.Moiety”  } |
| --- |
| **Molecule**  {  “name”: “UDP_GlcNAC”,  “moieties”: [{“py/id”: 13}, …],  “standardStates”: {“13C0”: [ [ “ribose[13C0]”, “glucose[13C0]”, “acetyl[13C0]”,  “uracil[13C0]”]], “13C10”: [ [“ribose[13C0]”, “glucose[13C6]”, “acetyl[13C2]”,  “uracil[13C2]”], [“ribose[13C5]”, “glucose[13C0]”, “acetyl[13C2]”,  “uracil[13C3]”], …], …},  “allStates”: [ “13C0”, “13C1”, “13C2”, “13C3”, “13C4”, “13C5”, “13C6”, “13C7”, “13C8”,  “13C9”, “13C10”, “13C11”, “13C12”, “13C13”, “13C14”, “13C15”, “13C16”, “13C17”],  “py/object”: “moiety_modeling.model.Molecule”  } |
| **Relationship**  {  “moiety”: {“py/id”: 13},  “moietyState”: “13C0”,  “varName”: “glucose[13C0]”,  “equivalentMoiety”: {“py/id”: 10},  “equivalentMoietyState”: “13C0”,  “equivalentVarName”: “ribose[13C0]”,  “multiplier”: 1,  “py/object”: “moiety_modeling.model.Relationship”  } |
| JSONized moiety model description. |
