# Supplementary material for: Moiety modeling framework for deriving moiety abundances from mass spectrometry measured isotopologues
Source: BMC Bioinformatics. 2019 Oct 28;20:524. doi: 10.1186/s12859-019-3096-7 (PMC6816163; doi:10.1186/s12859-019-3096-7)
Supplement: Supplementary file 6 — Additional file 6. The moiety_modeling package command line interface. [file 12859_2019_3096_MOESM6_ESM.docx]

| The moiety_modeling command-line interface.  Usage:  moiety_modeling –h \| --help  moiety_modeling --version  moiety_modeling modeling [--combinedData=<combined_jsonfile>] [--models=<models_jsonfile>] [--datasets=<datasets_jsonfile>] [--optimizations=<optimizations_jsonfile>] [--working=<working_dir>] [--repetition=<optim_count>] [--split] [--force] [--multiprocess] [--energyFunction=<function>] [--printOptimizationScripts]  moiety_modeling analyze optimization --a <optimzationPaths_txtfile> [--working=<working_dir]  moiety_modeling analyze optimization --s <optimzationResults_jsonfile> [--working=<working_dir]  moiety_modeling analyze rank <analysisPaths_txtfile> [--working=<working_dir>] [--rankCriteria=<rankCriteria>]  moiety_modeling analyze table <rankPaths_txtfile> [--working=<working_dir>]  moiety_modeling plot moiety <analysisResults_jsonfile> [--working=<working_dir>]  moiety_modeling plot isotopologue <analysisResults_jsonfile> [--working=<working_dir>]  Options:  -h, --help Show this screen.  --version Show version.  -combinedData JSON description file of the combined data (eg: models, datasets, optimization settings)  --models=<models_jsonfile> JSON description file of the moiety models.  --datasets=<datasets_jsonfile> JSON description file of the datasets.  --optimizaitons=<optimizaitons_jsonfile> JSON description file of the optimization setting.  --working=<working_dir> Alternative path to save the results.  --repetition=<optim_count> The number of optimization repetitions to perform [default: 100].  --split To split the datasets or not.  --force To force optimization process if error occurs.  --mulitprocess To perform with multiprocessing or not.  --printOptimizationScripts To print the optimization script or not.  --a To analyze a bunch of optimization results together with the path file.  --s To analyze a single moiety model optimization results.  --energyFunction=<function> The energy function for optimization [default: logDifference].  --optimzationSetting=<optimizationSetting> The optimization setting of the moiety modeling optimization.  --rankCriteria=<rankCriteria> The criteria for model ranking [default: AIC] |
| --- |
| The ‘moiety_modeling’ package command line interface. |
