# Supplementary material for: Moiety modeling framework for deriving moiety abundances from mass spectrometry measured isotopologues
Source: BMC Bioinformatics. 2019 Oct 28;20:524. doi: 10.1186/s12859-019-3096-7 (PMC6816163; doi:10.1186/s12859-019-3096-7)
Supplement: Supplementary file 7 — Additional file 7. Common patterns for using ‘SAGA’ module as a library. [file 12859_2019_3096_MOESM7_ESM.docx]

| **Table S1.** Common patterns for using ‘SAGA’ module as a library. | |
| --- | --- |
| **Usage** | **Example** |
| SAGA | saga = SAGA.SAGA(stepNumber=100, temperature=10, startTemperature=0.5, alpha=1, energyfunction=targertedEnergyFunction) |
|  | saga.addElmentDescriptions(SAGA.ElementDecription(low=0, high=1)) |
| Population | population = saga.optimize() |
| Guess | bestGuess = population.bestGuess |
