# Supplementary material for: Moiety modeling framework for deriving moiety abundances from mass spectrometry measured isotopologues
Source: BMC Bioinformatics. 2019 Oct 28;20:524. doi: 10.1186/s12859-019-3096-7 (PMC6816163; doi:10.1186/s12859-019-3096-7)
Supplement: Supplementary file 9 — Additional file 9. Model rank comparison between moiety_modeling and GAIMS. [file 12859_2019_3096_MOESM9_ESM.docx]

| **Table S2. Comparison of model rank between moiety_modeling (MM) and GAIMS.** | | | | |
| --- | --- | --- | --- | --- |
| **Models** | **MM**  **(AICc)** | **MM**  **(rank)** | **GAIMS**  **(AICc)** | **GAIMS**  **(rank)** |
| 7_G2R1A1U3_g5 | -420.0976537 | 1 | -432.945486 | 1 |
| 7_G1R2A1U3_r4 | -420.0527151 | 2 | -430.015253 | 4 |
| 6_G1R1A1U3 (expert-derived model) | -412.9252665 | 3 | -430.278915 | 3 |
| 7_G1R2A1U3_r3 | -404.6268861 | 4 | -415.017621 | 9 |
| 7_G2R1A1U3_g4 | -402.169066 | 5 | -411.415737 | 13 |
| 7_G1R1A2U3 | -401.750449 | 6 | -431.482325 | 2 |
| 8_G1R2A2U3_r3 | -401.7165641 | 7 | -410.303594 | 14 |
| 7_G1R1A1U4 | -398.409012 | 8 | -413.83319 | 11 |
| 7_G2R1A1U3_g1 | -397.4679486 | 9 | -411.45571 | 12 |
| 8_G2R1A2U3_g4 | -397.3625909 | 10 | -403.08689 | 23 |
| 7_G2R1A1U3_g2 | -395.7448889 | 11 | -410.05941 | 16 |
| 6_G1R1A1U3_r4 | -394.9864544 | 12 | -409.322715 | 17 |
| 8_G2R1A2U3_g1 | -392.2328434 | 13 | -406.83334 | 18 |
| 7_G1R2A1U3_r1 | -390.6784642 | 14 | -410.120097 | 15 |
| 6_G1R1A1U3_g5 | -389.6448147 | 15 | -414.14393 | 10 |
| 7_G1R1A1U3C1 | -386.9769515 | 16 | -403.173138 | 22 |
| 6_G1R1A1U3_u4 | -385.8790758 | 17 | -394.718199 | 27 |
| 6_G0R2A1U3_g3r2r3_g6r5 | -385.0778605 | 18 | -421.326047 | 6 |
| 8_G2R1A2U3_g5 | -381.7584641 | 19 | -400.135241 | 24 |
| 8_G1R2A2U3_r1 | -380.4285805 | 20 | -393.058791 | 28 |
| 8_G2R1A2U3_g2 | -373.003816 | 21 | -397.583784 | 25 |
| 7_G0R2A2U3_g3r2r3_g6r5 | -369.6325667 | 22 | -416.235838 | 8 |
| 8_G1R2A2U3_r4 | -363.4924892 | 23 | -391.901919 | 29 |
| 7_G2R1A1U3_g3 | -356.1215972 | 24 | -403.698136 | 21 |
| 7_G1R2A1U3_g3r2r3 | -355.7844636 | 25 | -405.161028 | 19 |
| 8_G1R1A2U3C1 | -355.0618972 | 26 | -390.434494 | 30 |
| 8_G1R2A2U3_r2 | -343.1849424 | 27 | -385.699884 | 31 |
| 7_G1R2A1U3_r2 | -343.0048832 | 28 | -404.046788 | 20 |
| 8_G2R1A2U3_g3 | -338.8111222 | 29 | -370.460991 | 34 |
| 8_G1R2A2U3_g3r2r3 | -333.5067784 | 30 | -424.625031 | 5 |
| 8_G1R2A2U3_g3r2r3_g6r5_g5 | -328.3546271 | 31 | -396.226273 | 26 |
| 6_G1R1A1U3_a1 | -320.4821446 | 32 | -330.249804 | 41 |
| 8_G1R2A2U3_r2r3 | -319.9764327 | 33 | -379.605343 | 32 |
| 9_G2R2A2U3_r2r3_g5 | -313.9283693 | 34 | -356.417739 | 36 |
| 9_G2R2A2U3_r2r3_g4 | -312.3476666 | 35 | -420.911799 | 7 |
| 9_G2R2A2U3_r2r3_g6r5_g3_g5 | -303.559521 | 36 | -367.532471 | 35 |
| 9_G2R2A2U3_r2r3_g1 | -282.1740027 | 37 | -336.285058 | 40 |
| 7_G0R3A1U3_g3r2r3_g6r5_g5r4 | -281.0076626 | 38 | -375.216445 | 33 |
| 9_G2R2A2U3_r2r3_g2 | -279.3550628 | 39 | -340.803226 | 38 |
| 9_G2R2A2U3_r2r3_g3 | -279.1101151 | 40 | -338.548888 | 39 |
| 7_G0R3A1U3_g3r2r3_g6r5_r4 | -243.859604 | 41 | -343.74261 | 37 |

Optimization settings: stepNumber’: 100000, ‘temperatureStepSize’: 100, ‘alpha’: 1, ‘crossoverRate’: 0.05, ‘mutationRate’: 3, ‘populationSize’: 20, ‘startTemperature’: 0.5, repetition=50, objective function=absolute difference.
