# Supplementary material for: Moiety modeling framework for deriving moiety abundances from mass spectrometry measured isotopologues
Source: BMC Bioinformatics. 2019 Oct 28;20:524. doi: 10.1186/s12859-019-3096-7 (PMC6816163; doi:10.1186/s12859-019-3096-7)
Supplement: Supplementary file 11 — Additional file 11. Comparison of optimized model parameters between moiety_modeing and GAIMS (Table). [file 12859_2019_3096_MOESM11_ESM.docx]

|  | | | | |
| --- | --- | --- | --- | --- |
| **Table S3. Comparison of optimized model parameters.** | | | | |
| **Model parameters** | **Moiety_modeling**  **Average** | **Moiety_modeling**  **SD** | **GAIMS**  **Average** | **GAIMS**  **SD** |
| DS0.acetyl.s0 | 0.500757 | 0.388488 | 0.657366 | 0.077231 |
| DS0.acetyl.s2 | 0.499243 | 0.388488 | 0.342634 | 0.077231 |
| DS0.uracil.s0 | 0.537884 | 0.362549 | 0.255905 | 0.077388 |
| DS0.uracil.s1 | 0.065682 | 0.017839 | 0.181486 | 0.010569 |
| DS0.uracil.s2 | 0.370573 | 0.353075 | 0.433916 | 0.066395 |
| DS0.uracil.s3 | 0.025860 | 0.023952 | 0.128693 | 0.020846 |
| DS0.glucose.s0 | 0.024036 | 0.012285 | 0.120516 | 0.040502 |
| DS0.glucose.s6 | 0.975964 | 0.012285 | 0.879484 | 0.040502 |
| DS0.ribose.s0 | 0.023717 | 0.014373 | 0.096301 | 0.03387 |
| DS0.ribose.s5 | 0.976283 | 0.014373 | 0.903699 | 0.03387 |
| DS1.acetyl.s0 | 0.444249 | 0.199454 | 0.564454 | 0.222067 |
| DS1.acetyl.s2 | 0.555751 | 0.199454 | 0.435546 | 0.222067 |
| DS1.uracil.s0 | 0.426045 | 0.156958 | 0.362826 | 0.185899 |
| DS1.uracil.s1 | 0.215096 | 0.034681 | 0.182222 | 0.030918 |
| DS1.uracil.s2 | 0.296197 | 0.134182 | 0.369483 | 0.160798 |
| DS1.uracil.s3 | 0.062663 | 0.055623 | 0.085469 | 0.05578 |
| DS1.glucose.s0 | 0.025567 | 0.022204 | 0.023816 | 0.017008 |
| DS1.glucose.s6 | 0.974433 | 0.022204 | 0.976184 | 0.017008 |
| DS1.ribose.s0 | 0.068814 | 0.020526 | 0.068395 | 0.022648 |
| DS1.ribose.s5 | 0.931186 | 0.020526 | 0.931605 | 0.022648 |
| DS2.acetyl.s0 | 0.576464 | 0.126600 | 0.609753 | 0.384462 |
| DS2.acetyl.s2 | 0.423536 | 0.126600 | 0.390247 | 0.384462 |
| DS2.uracil.s0 | 0.298522 | 0.111946 | 0.453468 | 0.366182 |
| DS2.uracil.s1 | 0.200751 | 0.019176 | 0.049118 | 0.012005 |
| DS2.uracil.s2 | 0.376906 | 0.102030 | 0.484239 | 0.362438 |
| DS2.uracil.s3 | 0.123821 | 0.025873 | 0.013175 | 0.007246 |
| DS2.glucose.s0 | 0.107953 | 0.042642 | 0.021585 | 0.011878 |
| DS2.glucose.s6 | 0.892047 | 0.042642 | 0.978415 | 0.011878 |
| DS2.ribose.s0 | 0.110747 | 0.038278 | 0.018137 | 0.010159 |
| DS2.ribose.s5 | 0.889253 | 0.038278 | 0.981863 | 0.010159 |
