# Supplementary material for: Moiety modeling framework for deriving moiety abundances from mass spectrometry measured isotopologues
Source: BMC Bioinformatics. 2019 Oct 28;20:524. doi: 10.1186/s12859-019-3096-7 (PMC6816163; doi:10.1186/s12859-019-3096-7)
Supplement: Supplementary file 12 — Additional file 12. Comparison of model rank between different repetitions. [file 12859_2019_3096_MOESM12_ESM.docx]

| **Table S4. Comparison of model rank of two repetitions.** | | | | |
| --- | --- | --- | --- | --- |
| **Models** | **AICc(1^st^)** | **Rank(1^st^)** | **AICc(2^ed^)** | **Rank(2^ed^)** |
| 6_G1R1A1U3 (expert-derived model) | -229.2918 | 1 | -228.85836 | 1 |
| 6_G1R1A1U3_r4 | -227.5208 | 2 | -227.11248 | 2 |
| 6_G1R1A1U3_u4 | -225.0006 | 3 | -224.99505 | 3 |
| 6_G0R2A1U3_g3r2r3_g6r5 | -223.1633 | 4 | -223.88568 | 4 |
| 6_G1R1A1U3_g5 | -215.9565 | 5 | -215.6796 | 5 |
| 7_G1R2A1U3_r1 | -212.4727 | 6 | -212.67281 | 6 |
| 7_G2R1A1U3_g1 | -212.1217 | 7 | -212.22038 | 7 |
| 7_G1R2A1U3_r3 | -210.964 | 8 | -210.36248 | 9 |
| 7_G1R1A2U3 | -210.0952 | 9 | -210.77011 | 8 |
| 7_G2R1A1U3_g5 | -208.1346 | 10 | -208.51863 | 10 |
| 7_G1R2A1U3_g3r2r3 | -207.6523 | 11 | -207.18463 | 12 |
| 7_G1R2A1U3_r2 | -207.4187 | 12 | -207.46405 | 11 |
| 7_G2R1A1U3_g4 | -206.643 | 13 | -206.72506 | 13 |
| 7_G2R1A1U3_g2 | -206.5609 | 14 | -206.14537 | 14 |
| 7_G0R2A2U3_g3r2r3_g6r5 | -205.0569 | 15 | -204.286 | 16 |
| 7_G2R1A1U3_g3 | -204.8797 | 16 | -205.76382 | 15 |
| 7_G0R3A1U3_g3r2r3_g6r5_g5r4 | -204.2729 | 17 | -204.10886 | 17 |
| 7_G1R1A1U4 | -203.371 | 18 | -202.81321 | 18 |
| 7_G1R2A1U3_r4 | -202.6782 | 19 | -202.39866 | 19 |
| 6_G1R1A1U3_a1 | -199.556 | 20 | -199.45647 | 20 |
| 8_G2R1A2U3_g1 | -195.9713 | 21 | -194.84803 | 22 |
| 7_G1R1A1U3C1 | -195.5788 | 22 | -195.77553 | 21 |
| 8_G1R2A2U3_r1 | -195.4893 | 23 | -194.15639 | 23 |
| 7_G0R3A1U3_g3r2r3_g6r5_r4 | -192.498 | 24 | -192.0369 | 24 |
| 8_G1R2A2U3_r2r3 | -187.3342 | 25 | -186.16034 | 26 |
| 8_G1R2A2U3_r3 | -186.881 | 26 | -186.76368 | 25 |
| 8_G2R1A2U3_g5 | -186.2693 | 27 | -186.1201 | 27 |
| 8_G1R2A2U3_r2 | -186.2562 | 28 | -185.16902 | 29 |
| 8_G2R1A2U3_g2 | -185.6112 | 29 | -185.43328 | 28 |
| 8_G2R1A2U3_g4 | -184.9444 | 30 | -185.10249 | 30 |
| 8_G1R2A2U3_g3r2r3 | -184.2929 | 31 | -184.56788 | 31 |
| 8_G1R2A2U3_g3r2r3_g6r5_g5 | -183.2154 | 32 | -184.09402 | 33 |
| 8_G2R1A2U3_g3 | -183.1467 | 33 | -184.19673 | 32 |
| 8_G1R2A2U3_r4 | -182.1334 | 34 | -180.97774 | 34 |
| 8_G1R1A2U3C1 | -177.5013 | 35 | -176.80891 | 35 |
| 9_G2R2A2U3_r2r3_g1 | -170.3323 | 36 | -170.01241 | 36 |
| 9_G2R2A2U3_r2r3_g2 | -161.577 | 37 | -161.71805 | 37 |
| 9_G2R2A2U3_r2r3_g3 | -160.7823 | 38 | -161.07108 | 39 |
| 9_G2R2A2U3_r2r3_g6r5_g3_g5 | -160.6917 | 39 | -161.57823 | 38 |
| 9_G2R2A2U3_r2r3_g4 | -160.45 | 40 | -160.10174 | 40 |
| 9_G2R2A2U3_r2r3_g5 | -158.8733 | 41 | -159.43149 | 41 |

Optimization settings: method = ’SAGA’, SAGA_parameters = {‘stepNumber’: 100000, ‘temperatureStepSize’: 100, ‘alpha’: 1, ‘crossoverRate’: 0.05, ‘mutationRate’: 3, ‘populationSize’: 20, ‘startTemperature’: 0.5}, repetition=100, split, objective function=log difference.
