# Supplementary material for: Moiety modeling framework for deriving moiety abundances from mass spectrometry measured isotopologues
Source: BMC Bioinformatics. 2019 Oct 28;20:524. doi: 10.1186/s12859-019-3096-7 (PMC6816163; doi:10.1186/s12859-019-3096-7)
Supplement: Supplementary file 13 — Additional file 13. Multi-tracer optimization results for simulated datasets. [file 12859_2019_3096_MOESM13_ESM.docx]

**
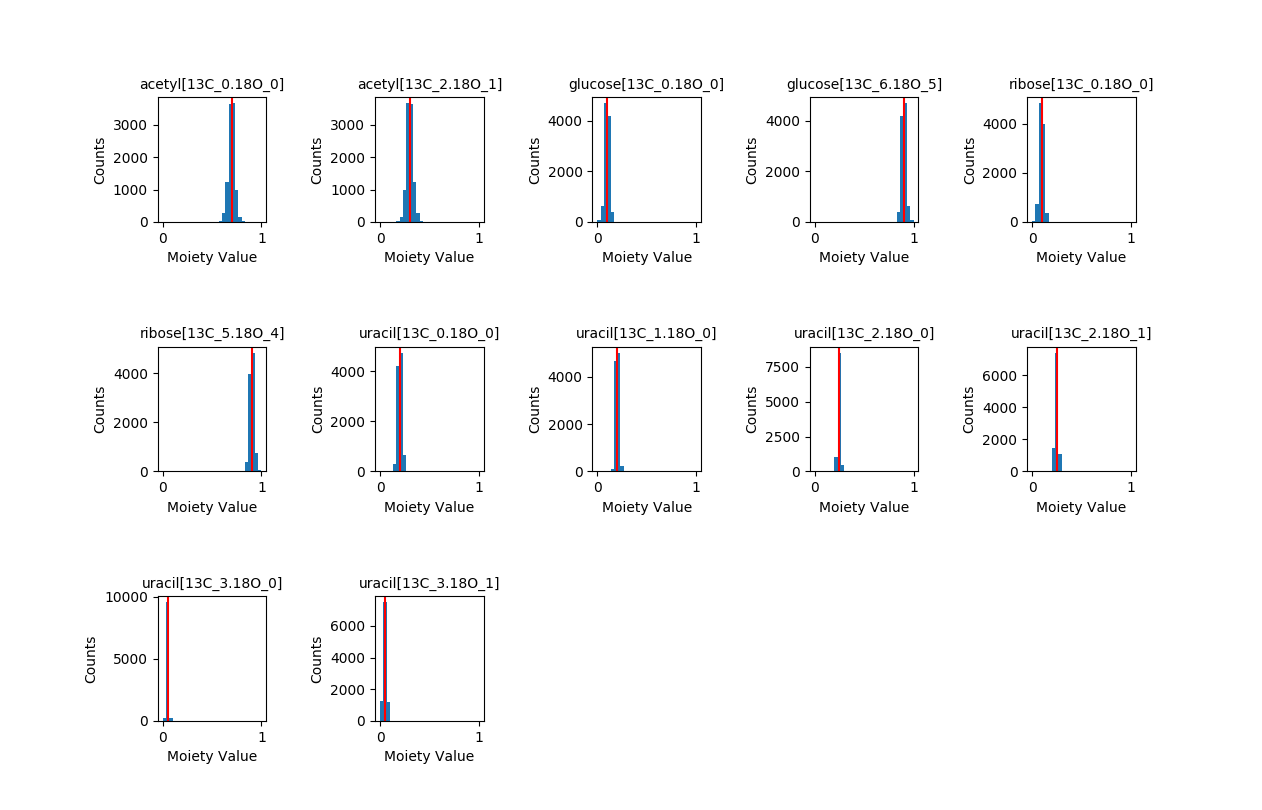
Multi-tracer Optimization Results (additive error σ = 0.001, estimated error propagation = 0.02)**

**
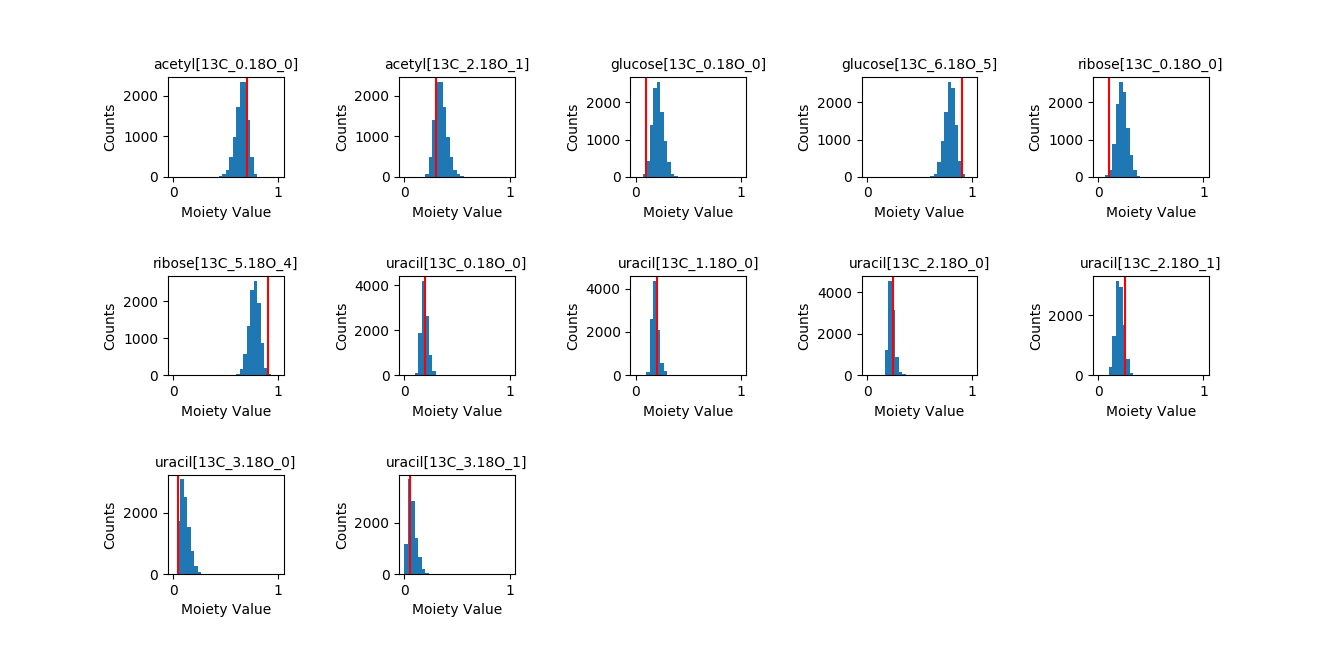
Multi-tracer Optimization Results (additive error σ = 0.01, estimated error propagation = 0.73)**

**
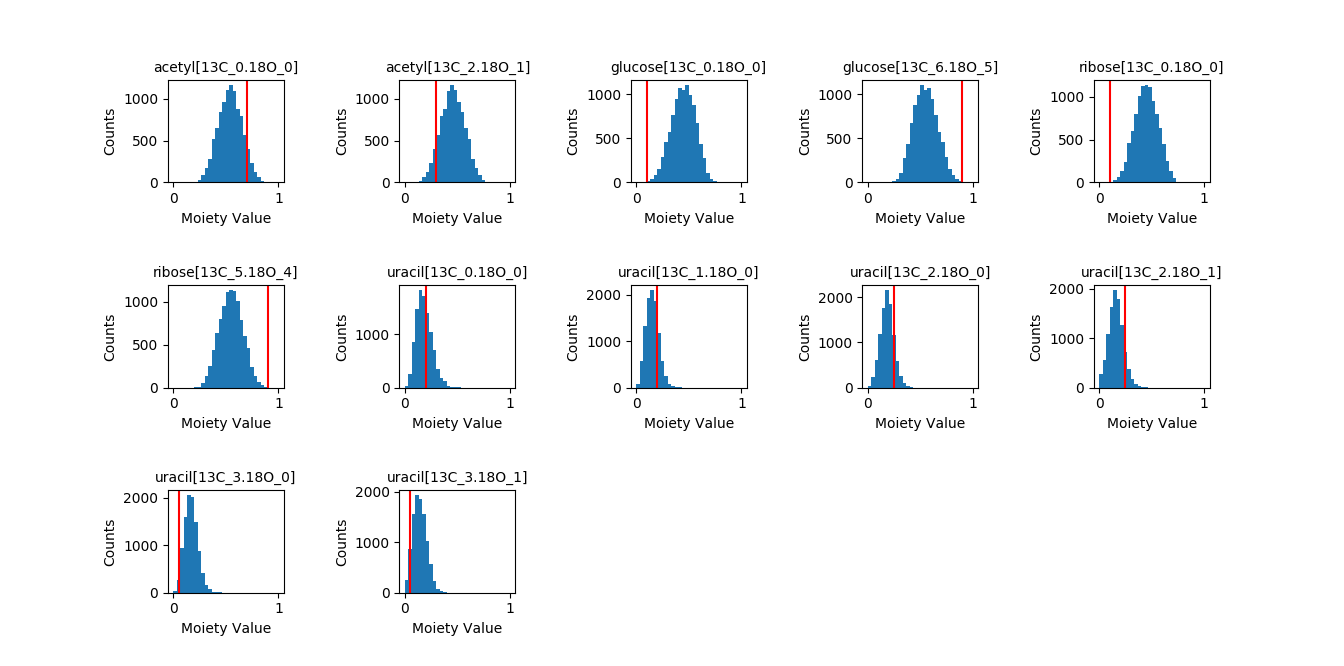
Multi-tracer Optimization Results (additive error σ = 0.1, estimated error propagation = 8.97)**
