# Supplementary material for: Moiety modeling framework for deriving moiety abundances from mass spectrometry measured isotopologues
Source: BMC Bioinformatics. 2019 Oct 28;20:524. doi: 10.1186/s12859-019-3096-7 (PMC6816163; doi:10.1186/s12859-019-3096-7)
Supplement: Supplementary file 14 [file 12859_2019_3096_MOESM10_ESM.docx]

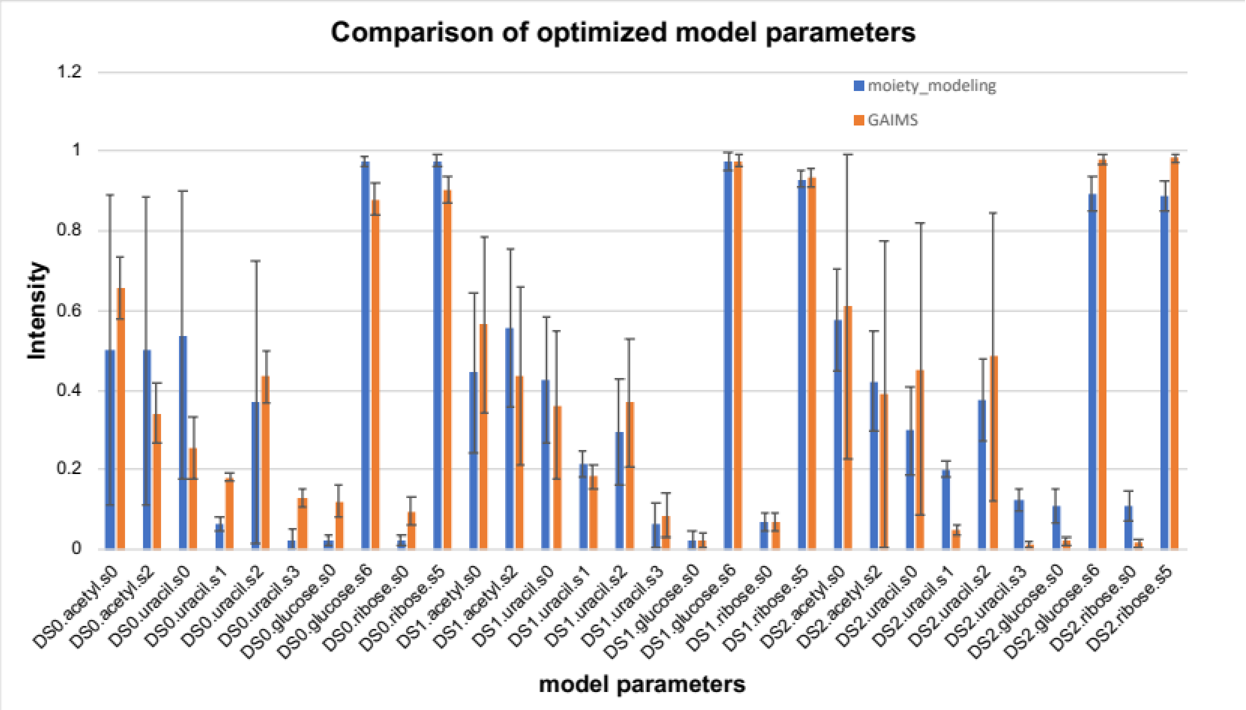

Comparison of optimized model parameters
moiety_modeling
GAIMS
Intensity
1.2
1
0.8
0.6
0.4
0.2
0
DS0.acetyl.s0
DS0.acetyl.s2
DS0.uracil.s0
DS0.uracil.s1
DS0.uracil.s2
DS0.uracil.s3
DS0.glucose.s0
DS0.glucose.s6
DS0.ribose.s0
DS0.ribose.s5
DS1.acetyl.s0
DS1.acetyl.s2
DS1.uracil.s0
DS1.uracil.s1
DS1.uracil.s2
DS1.uracil.s3
DS1.glucose.s0
DS1.glucose.s6
DS1.ribose.s0
DS1.ribose.s5
DS2.acetyl.s0
DS2.acetyl.s2
DS2.uracil.s0
DS2.uracil.s1
DS2.uracil.s2
DS2.uracil.s3
DS2.glucose.s0
DS2.glucose.s6
DS2.ribose.s0
DS2.ribose.s5
model parameters
